# The membrane-bound and soluble form of melanotransferrin function independently in the diagnosis and targeted therapy of lung cancer

**DOI:** 10.1038/s41419-020-03124-2

**Published:** 2020-10-30

**Authors:** Yuanyuan Lei, Zhiliang Lu, Jianbing Huang, Ruochuan Zang, Yun Che, Shuangshuang Mao, Lingling Fang, Chengming Liu, Xinfeng Wang, Sufei Zheng, Nan Sun, Jie He

**Affiliations:** National Cancer Center/National Clinical Research Center for Cancer/Cancer Hospital, Chinese Academy of Medical Sciences and Peking Union Medical College, Beijing, China

**Keywords:** Non-small-cell lung cancer, Tumour biomarkers

## Abstract

Melanotransferrin (MFI2) is a newly identified tumor-associated protein, which consists of two forms of proteins, membrane-bound (mMFI2) and secretory (sMFI2). However, little is known about the expression pattern and their relevance in lung cancer. Here, we found that both two forms of MFI2 are highly expressed in lung cancer. The expression of MFI2 in lung cancer was detected by using the public database and qRT-PCR. Overexpression and knockdown cell lines and recombinant sMFI2 protein were used to study the function of mMFI2 and sMFI2. RNA-seq, protein chip, ChIP assay, Immunoprecipitation, ELISA, and immunofluorescence were used to study the molecular biological mechanism of mMFI2 and sMFI2. We found that mMFI2 promoted the expression of EMT’s common marker N-cadherin by downregulating the transcription factor KLI4, which in turn promoted tumor metastasis; sMFI2 could promote the metastasis of autologous tumor cells in an autocrine manner but the mechanism is different from that of mMFI2. In addition, sMFI2 was proved could inhibit the migration of vascular endothelial cells and subsequently enhance angiogenic responses in a paracrine manner. We propose that the expressions and functions of the two forms of MFI2 in lung cancer are relatively independent. Specifically, mMFI2 was a potential lung cancer therapeutic target, while sMFI2 was highly enriched in advanced lung cancer, and could be used as a tumor staging index.

## Introduction

Lung cancer is the most common malignant tumor in the world^1^. In China, more than 85% of lung cancer patients are non-small cell lung cancer (NSCLC)^2^. Although the diagnosis and treatment techniques for lung cancer have been greatly improved in recent years, the 5-year survival rate is only about 10–20%^3^. On the one hand, most lung cancer patients are diagnosed at the advanced stage of cancer, lost the best treatment opportunity; on the other hand, clinically available conventional chemotherapy and existing targeted therapies have limited efficacy, and a higher proportion of postoperative recurrence and metastasis also affects their efficacy. Therefore, researching effective therapeutic targets and finding diagnostic and prognostic markers with high sensitivity and specificity are the key to improve the prognosis of patients with lung cancer.

Cell surface proteins carry a variety of essential cellular functions, including communication between cells and cells, signal transduction, and transmembrane transport of substances. The expression profiles of cell membrane surface proteins vary greatly between cells, reflecting pathological progression^4^. Currently, a variety of cancer-related cell surface protein disorders including changes in expression levels or protein variants have been widely reported^5,6^. Therefore, studying proteins specifically expressed on the membrane of cancer cells provides new ideas for molecular typing of NSCLC and exploring new effective therapeutic targets.

Melanotransferrin (hereinafter abbreviated MFI2) is called melanin transferrin, first discovered in glycoprotein on the surface of melanoma cells, in two forms and two different length transcripts, one (the longer) bound to the membrane (mMFI2) and the other (the shorter) secreted to the peripheral environment (sMFI2) such as blood and cell supernatant. The protein name is derived from its sequence similar to the transferrin superfamily and its ability to bind iron, but studies have shown that the MFI2 protein has only one iron-binding site at the N-terminus. Studies showed the mice overexpressing MFI2 had no significant changes in phenotype except for the decrease in hemoglobin, indicating that it is not a typical transferrin^7,8^. MFI2 has only a small amount of expression in normal tissues, but a large amount of expression in tumor tissues and embryo tissues. Research on MFI2 is mainly in melanoma which is associated with tumor metastasis and angiogenesis. The level of MFI2 in peripheral blood of patients with colorectal cancer is also elevated^9,10^. Although the above reports focus on the expression of MFI2 in certain tumors, there is no detailed study of mMFI2 and sMFI2 in NSCLC, moreover, the relationship between mMFI2 and sMFI2 is unknown.

Here, we constructed a stable cell line with high or low expression of MFI2. At the same time, we purchased the recombinant sMFI2 protein, and conducted an in-depth study on the function and mechanism of membrane-bound and secreted MFI2 in lung cancer. We found that although these two molecules have a large degree of overlap at the genetic level, the effect was somewhat different.

## Results

### MFI2 is significantly upregulated in lung cancer tissues and cell lines

**Fig. 1:**
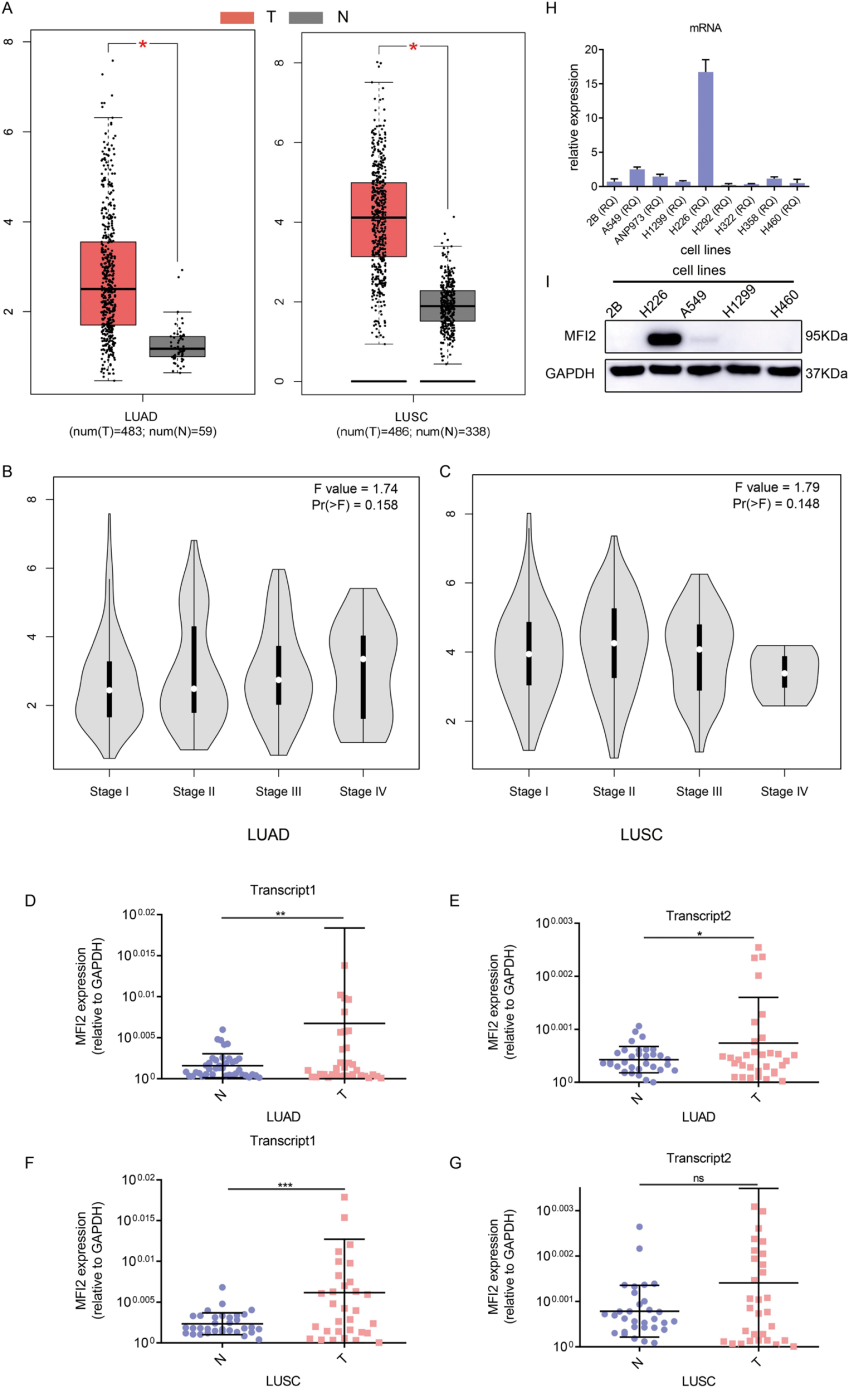
MFI2 is significantly upregulated in lung cancer tissues and cell lines. **A** Expression of MFI2 in GEPIA databases. The left is the expression of 483 lung adenocarcinoma tissues and 59 normal tissues, and the right side is the expression of 486 lung squamous cell carcinoma tissues and 338 normal tissues. LUAD, lung adenocarcinoma, LUSC, lung squamous cell carcinoma. **B**, **C** Expression of MFI2 in different stages of lung adenocarcinoma and lung squamous cell carcinoma. **D**–**G** The expression of two transcripts of MFI2, which were normalized to that of GAPDH, in fresh-frozen samples of Lung adenocarcinoma and lung squamous cell carcinoma, as determined by RT-qPCR. **H**, **I** Detection of MFI2 expression in common cell lines by RT-qPCR and western blot. Data are presented as the mean ± SD, *n* = 3. *p < 0.05, **p < 0.01, ***p < 0.001. ns means no significance.

We first analyzed the data in the public databases GEPIA. In 485 cases of lung adenocarcinoma (LUAD) and 59 cases of normal lung tissue, the expression level of MFI2 in adenocarcinoma was significantly higher than that in normal lung tissue, *p* value <0.01 (Fig. 1A), and the expression levels in late stage were higher than in early stage (Fig. 1B); similarly, in 486 cases of lung squamous cell carcinoma (LUSC) and 338 normal lung tissues, the expression of MFI2 in lung cancer was significantly higher than that in normal tissues, *p* value <0.01 (Fig. 1A). However, there was no difference in the expression of MFI2 in different lung cancer stages (Fig. 1C). The above mentioned that mMFI2 and sMFI2 encoded by two different transcripts differ greatly in molecular function. Thus, we next wanted to investigate the detailed difference between the expression levels of these two transcripts in lung cancer. Forty cases of lung adenocarcinoma and 40 cases of lung squamous cell carcinoma and their corresponding adjacent tissues of frozen tissues were analyzed by qPCR. The results showed that both two transcripts had high expression in lung adenocarcinoma and lung squamous cell carcinoma, *p* value <0.05 (Fig. 1D–G). Moreover, we analyzed the expression of MFI2 in common lung cancer cell lines. Compared with normal cells, expression of MFI2 was high in H226, intermediate in A549, but clearly low in H1299 and H460 (Fig. 1H and I).

### Membrane-bound MFI2 (mMFI2) significantly promotes tumor cell migration and invasion and inhibits cisplatin-induced apoptosis

**Fig. 2:**
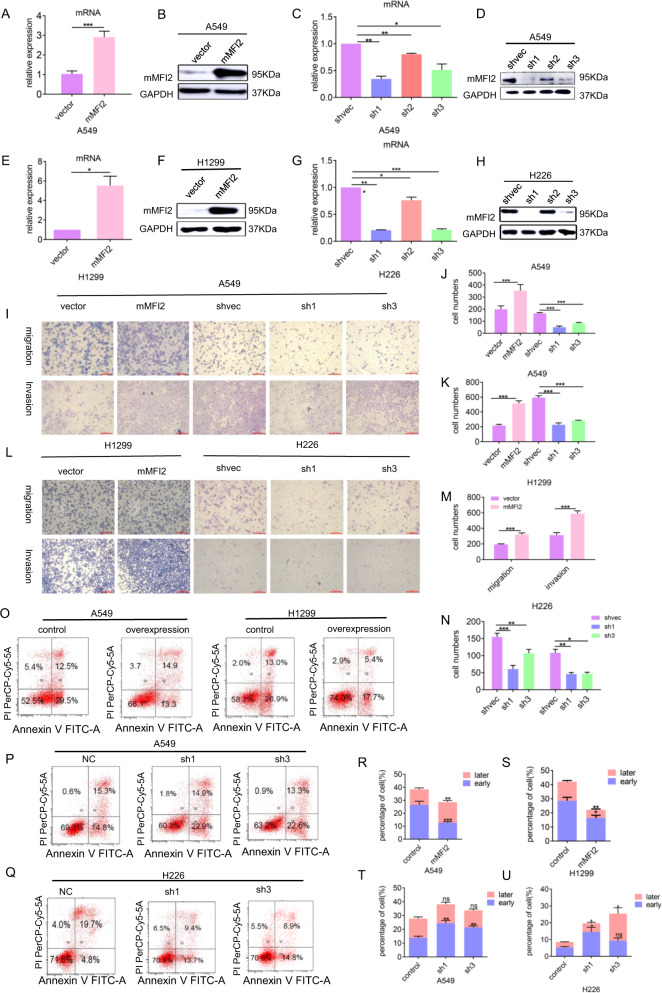
mMFI2 significantly promotes tumor cell migration and invasion, and inhibits cisplatin-induced apoptosis. **A**, **B** and **E**, **F** mMFI2 expression levels in stable overexpression cell clones A549 and H1299, as determined by RT-qPCR and western blot. Bar graph showing the results of the RT-qPCR analysis of mMFI2 expression. **C**, **D** and **G**, **H** mMFI2 expression levels in stable knockdown cell clones A549 and H226, as determined by RT-qPCR and western blot. mMFI2 expression was significantly downregulated in the knockdown clones. Bar graph showed the results of the RT-qPCR analysis of mMFI2 expression. **I**–**N** The migration and invasion ability of mMFI2 overexpression and knockdown cell lines, as well as that of mock-vehicle control-transfected cells, was detected by transwell assay. Bar chart showed the difference in cell numbers between groups. **O**–**U** The A549, H226, and H1299 cell clones in which mMFI2 were stably overexpressed or knocked down were seeded in 6 cm plates, treated with cisplatin, and then cultured for 24 h. Apoptosis was measured with FACS-based annexin-V/PI double staining. The histogram showed the specific proportion of early and late apoptosis in each cell clone. Data are presented as the mean ± SD, *n* = 3. *p < 0.05, **p < 0.01, ***p < 0.001. ns means no significance.

We selected cell line H1299 with low expression of endogenous mMFI2, transfected lentiviral plasmid to construct a stable mMFI2 overexpressing cell clone, cell line H226 with high expression of endogenous mMFI2 to construct a stable knockdown cell clone, and for the cell line A549 with endogenous mMFI2 expression at neutral level, we transfected mMFI2 overexpression and knockdown plasmids, respectively. The functional experiments of cells in which mMFI2 was overexpressed (Fig. 2A, B and E, F) or silenced (Fig. 2C, D and G, H) demonstrated that mMFI2 had no effect on cell growth or cycle (Supplementary Figs. S1A and B), consistent with the study of genetic modification in melanoma^8^, but it could significantly affect the process of cell migration and invasion. Overexpression of mMFI2 significantly promoted migration and invasion of A549 and H1299 cell lines (Fig. 2I–M) and knockdown of mMFI2 significantly inhibited migration and invasion of A549 and H226 cell lines (Fig. 2I–N). Meanwhile, we were surprised to find that this protein could inhibit cisplatin-induced apoptosis, especially early apoptosis (Fig. 2O–U). In order to explain this phenomenon, we screened a protein chip containing 35 apoptosis-related key proteins in mMFI2 overexpressing and non-overexpressing cell lines. The results showed that the endogenous apoptotic pathway did have been activated (Supplementary Figs. S2A and B) and mMFI2 might be related to the activation of a series of stress-related pathways triggered by its own membrane protein properties, which have not been reported. However, we did not know that the main function of MFI2 was to promote metastasis or inhibit apoptosis. Further confirmation by the next experiment was needed.

### mMFI2 promotes expression of EMT-related marker N-cadherin by downregulating KLF4

**Fig. 3:**
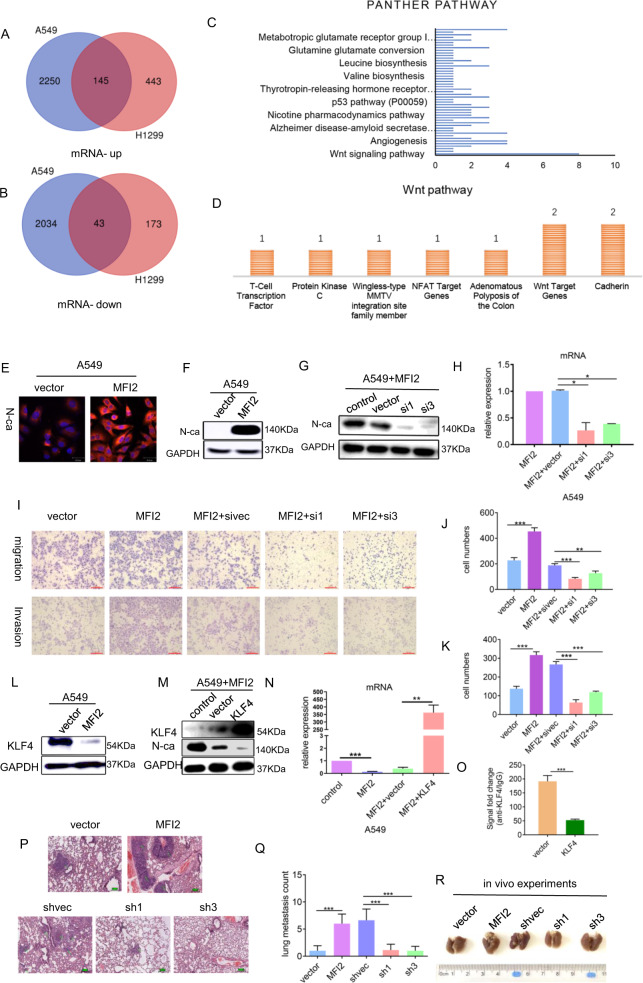
mMFI2 promotes expression of EMT-related marker N-cadherin by downregulating KLF4. **A**, **B** Venn diagram indicated the common upregulated and downregulated genes in the mMFI2 overexpressing cell lines A549 and H1299. **C**, **D** The differentially expressed genes were significantly enriched by the PANTHER pathway analysis, and the differential gene in the Wnt pathway was shown in the figure below. The cadherin family changed significantly. **E**, **F** Immunofluorescence and western blot showed that N-cadherin was highly expressed in cell lines overexpressing mMFI2. **G**, **H** Western blot and RT-PCR verified the siRNA knockdown efficiency of N-cadherin in cell line A549. **I**–**K** Changes in migration and invasion of cells with knockdown of N-cadherin. **L** Western blot confirmed the low expression of KLF4 in cell lines overexpressing mMFI2. **M**, **N** Western blot and RT-PCR confirmed the expression efficiency of KLF4 and the expression of N-cadherin after KLF4 overexpression. **O** chromatin immunoprecipitation demonstrated reduced binding of KLF2 to N-cadherin promoter in overexpressing mMFI2 cell lines. **P** Hematoxylin and eosin-stained images of lung tissues. 10 mice per group, a total of 50 mice were used. **Q**, **R** Representative images and the number of metastatic nodules in the lung tissues isolated from mice injected with A549 cell clones via the tail vein. The tumor counting method was as follows: lung tissue was sliced according to the largest cross section, and then HE staining was performed to determine the presence or absence of tumors. The number of lung metastases was determined by the number of tumors with modified cross section. Data are presented as the mean ± SD, *n* = 3. *p < 0.05, **p < 0.01, ***p < 0.001.

We performed transcriptome sequencing on cell line H1299 and H226 with or without high expression of mMFI2. By differential gene enrichment analysis (fold change >2; *p* value <0.05), we found 145 genes were simultaneously upregulated in two cell lines and 43 genes were simultaneously downregulated (Fig. 3A and B). Screened differential genes were imported into the PANTHER tool (http://pantherdb.org/) for pathway enrichment analysis. The Wnt signaling pathway was significantly enriched (Fig. 3C) and the most changed gene in this pathway was the cadherin family (Fig. 3D). Later immunofluorescence and western blot confirmed that EMT-related important gene N-cadherin was highly expressed in mMFI2 overexpressing cell clones (Fig. 3E and F). In order to prove the regulatory effect of mMFI2 on N-cadherin, we deliberately knocked down N-cadherin to observe whether the biological function of mMFI2 changed. The knockdown efficiency was shown in Fig. 3G and H. We found that the increased cell migration and invasion capacity by mMFI2 was attenuated by knockdown of N-cadherin expression (Fig. 3I–K). Based on the data, it could be inferred that the upregulation of N-cadherin was first at the mRNA level, and some transcription factors may affect its transcription process. By reviewing the literature^11^ and database Gene Cards and JASPAR predictions, we found that the transcription factor KLF4 might bind to the promoter region of N-cadherin and inhibit the transcription of N-cadherin. KLF4 is a common transcription factor that inhibits the transcription of multiple tumorigenic genes, and its high expression is associated with poor prognosis in many tumors^12–15^. Moreover, studies have found that KLF4 accumulation in the nucleus of lung cancer has a worse prognosis than cytoplasmic accumulation^16^. Our experiments confirmed that KLF4 did show low expression in cells overexpressing mMFI2 (Fig. 3L). To confirm the hypothesis above, we constructed a KLF4 overexpression plasmid that was transiently transfected into the cell line overexpressing mMFI2. After overexpression of KLF4, the upregulation of N-cadherin by mMFI2 was significantly reduced (Fig. 3M and N). The results of chromatin immunoprecipitation confirmed that the binding of KLF4 to N-cadherin decreased after overexpression of mMFI2 (Fig. 3O).

To verify the ability of mMFI2 to promote metastasis in vivo, A549 cells with knockdown or overexpression of MFI2, and control cells were injected into the blood of NOD/SCID mice by tail vein in an amount of 2 × 10^6^/mouse. H&E-stained images of lung tissue specimens are shown in Fig. 3P. The mouse overexpressing mMFI2 had more lung metastases than the control group, but the mouse with mMFI2 knockdown showed a significant decrease in metastatic nodules relative to the control group (Fig. 3Q and R).

### sMFI2 affects the migration and invasion of its own cells through the autocrine pathway, different form mMFI2

**Fig. 4:**
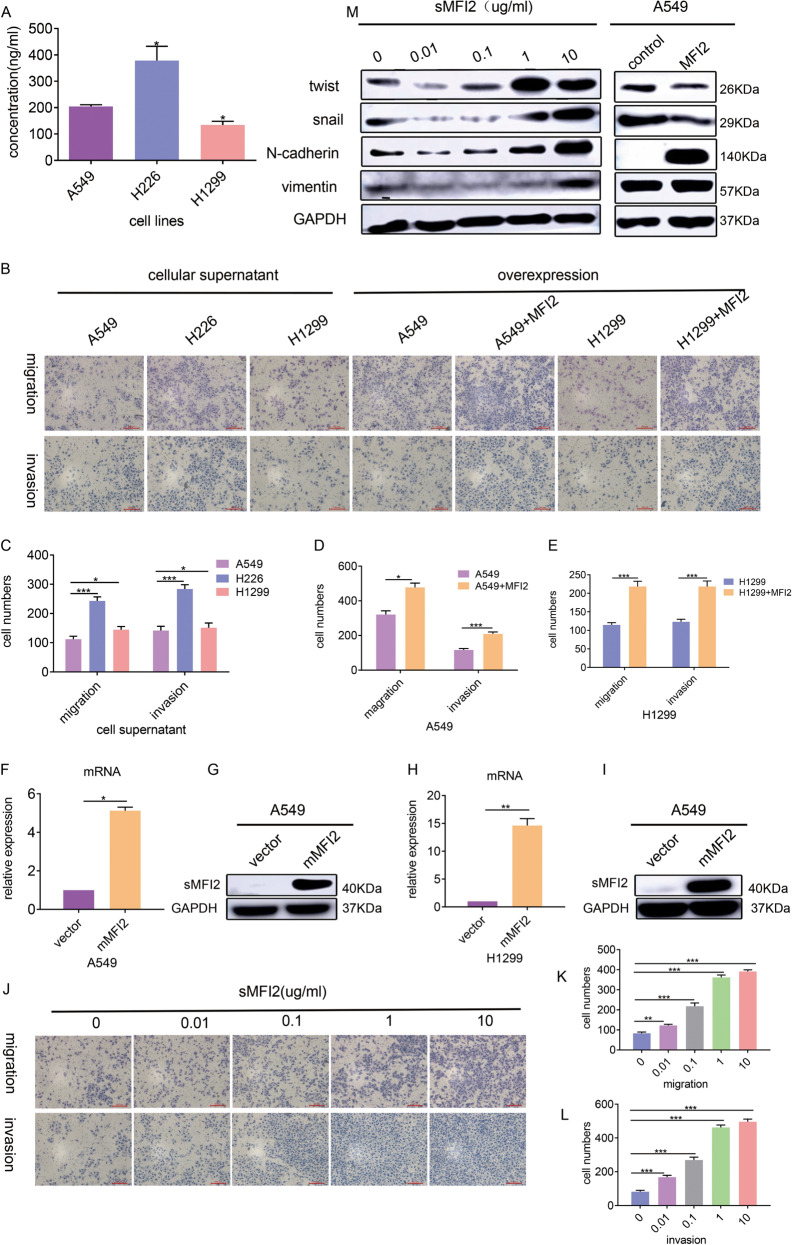
sMFI2 affects the migration and invasion of its own cells through the autocrine pathway, but not the same as the mMFI2. **A** Histogram showed sMFI2 content of cell culture supernatants in wild-type A549, H226, and H1299 detected by ELISA. **B**–**E** Changes in migration and invasion ability of wild-type cell line A549 when it was co-cultured with cell supernatant of its own and other wild-type cell lines H226 and H1299 and cells stably transfected with sMFI2. Bar graph showing changes in the number of cells in each group. **F**–**I** Western blot and RT-PCR verified the sMFI2 overexpression efficiency in cell line A549. **J**–**L** Changes of migration and invasion ability of wild-type cell line A549 after co-culturing with different concentrations of sMFI2. **M** Western blot showed changes in wild-type cell line A549 in EMT-related markers after overexpression of sMFI2 or co-culturing with different concentrations of sMFI2. Data are presented as the mean ± SD, *n* = 3. *p < 0.05, **p < 0.01, ***p < 0.001.

The exact function of sMFI2 is highly controversial. Some reports suggested that sMFI2 had a tumor-promoting effect^17^, but others had also shown that sMFI2 is a tumor suppressor gene^18–20^. We first detected sMFI2 in the cell supernatants of H226, A549, and H1299 by ELISA and found that there was a certain amount of sMFI2 (Fig. 4A). Then we explored whether sMFI2 could promote tumor cell metastasis like mMFI2, as they might only differ in structure by a GPI-anchored protein^21^. we concentrated the above cell supernatant and co-cultured it with wild-type A549. The results showed that the cell supernatants with high levels of sMFI2 had strong ability of promoting the migration of A549 cells (Fig. 4B and C). Although mMFI2 and sMFI2 were encoded by different transcripts of the same gene, it was not excluded that mMFI2 also could be secreted to some extent. We then doubted whether the MFI2 protein in the cell supernatant could be a mixture. In order to observe the effect of sMFI2 alone, we constructed cell clones A549 and H1299 stably overexpressing the sMFI2 gene (Fig. 4F–I). Similarly, the cell supernatants of the two overexpressed cell lines and the corresponding control group were collected and concentrated, and co-cultured with wild-type A549. The overexpression group, compared with the control group, had stronger ability to promote migration of wild-type A549 (Fig. 4B, D, and E). The above experiments confirmed that sMFI2 could promote the migration of autologous tumor cells in an autocrine manner.

Furthermore, in order to exclude the influence of other components of the supernatant, we purchased the recombinant sMFI2 protein and diluted it to different concentrations to co-culture with wild-type A549. As the concentration of recombinant sMFI2 increased, the migration-promoting ability became stronger, and the maximum effect was at 1 μg/ml (Fig. 4J–L). In order to have a general understanding of the mechanism of recombinant sMFI2 in promoting migration, we analyzed the changes of common EMT markers in wild-type A549 after treatment at different concentrations of sMFI2. Unlike the mMFI2, which only affected expression of N-cadherin, sMFI2 triggered upregulation of a series of EMT markers. For example, twist, snail, N-cadherin, and vimentin were obviously upregulated (Fig. 4M). The above experiments demonstrated that sMFI2 could promote the metastasis of autologous cells in the same way as mMFI2 in an autocrine manner, but the mechanism of promoting metastasis was different from mMFI2.

### sMFI2 inhibits vascular endothelial cell migration and angiogenesis via a paracrine pathway

**Fig. 5:**
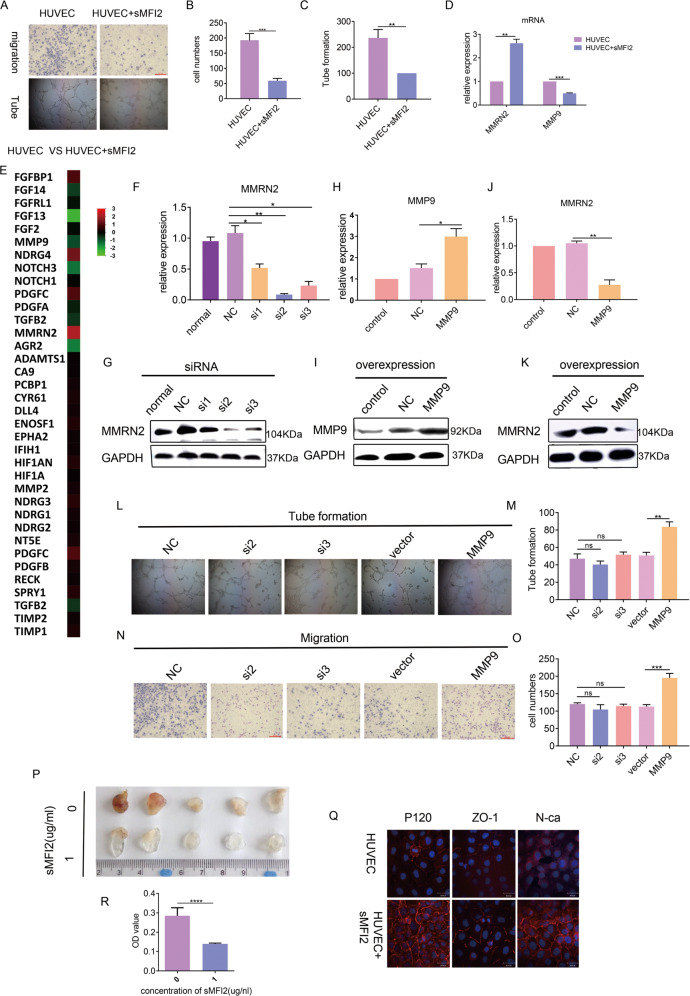
sMFI2 inhibits vascular endothelial cell migration and angiogenesis via a paracrine pathway without affecting vascular permeability. **A**–**C** Changes in migration ability and angiogenic ability of vascular endothelial cell HUVEC after co-culturing with recombinant sMFI2. **D** RT-PCR analysis of the expression changes of MMRN2 and MMP9 genes in HUVECs with sMFI2 co-culture or non-co-culture. **E** Heat map showed differential expression of common angiogenesis-related genes in HUVEC with sMFI2 co-cultured or non-co-cultured groups. The screening criterion was fold change ≥2, *p* < 0.05. **F**, **G** RT-PCR and western blot showed the knockdown efficiency of MMRN2 after transient transfection of siRNA. **H**, **I** RT-PCR and western blot showed overexpression efficiency of MMP9 after transient transfection of overexpressing plasmid. **J**, **K** RT-PCR and western blot showed changes in the expression of MMRN2 after overexpression of MMP9. **L**–**O** Changes in migration ability of vascular endothelial cells and tubule formation ability after knockdown of MMRN2 or overexpression of MMP9. Image taken under a ×100 microscope. Histogram showed the number of HUVEC cells and the quantification of vascularization between groups. **P**–**R** Representative pictures of angiogenesis in HUVEC under co-culture or non-co-culture conditions with recombinant sMFI2. Bar graph showed the relative content of hemoglobin between two groups. **Q** Immunofluorescence showed changes in intercellular junction markers P120, ZO-1, and N-cadherin of HUVEC under co-culture and non-co-culture conditions with sMFI2. Data are presented as the mean ± SD, *n* = 3. *p < 0.05, **p < 0.01, ***p < 0.001.

As a secreted protein, paracrine function often coexists with autocrine. Current reports on sMFI2 rarely focus on its autocrine function, as it can regulate the function of surrounding stromal cell and endothelial cells in a paracrine manner^17,22^. As mentioned above, the results of this part of the study are currently controversial and there are no detailed mechanisms for research. According to the maximum stimulation amount of 1 μg/ml, we co-cultured recombinant sMF2 with umbilical vein endothelial cells HUVEC. Compared with the control group, the endothelial cell migration ability and tubule formation ability of the co-culture group were significantly weakened (Fig. 5A–C). In order to explore the specific mechanism, we performed transcriptome sequencing on HUVEC with or without co-culturing with sMFI2. Differential expression gene analysis showed that EMT-related genes were not significantly enriched (data not shown). Then, we focused on angiogenesis-related genes. Enrichment analysis of common angiogenesis-related genes revealed significant upregulation of MMRN2 (Fig. 5E). qPCR confirmed that MMRN2 did have high expression in the co-culture group (Fig. 5D), demonstrating the accuracy of RNA-seq data. According to reports in the literature, MMRN2 was considered to be a factor that inhibits angiogenesis^23,24^. We knocked down MMRN2 by siRNA (Fig. 5F and G) The results also showed that HUVEC cells treated with recombinant sMFI2 did not show reduced angiogenic capacity after siRNA knockdown of MMRN2 expression (Fig. 5L–M). This indicated that recombinant sMFI2 did inhibit angiogenesis via MMRN2. MMRN2 is a secreted protein, it is regulated by the metalloproteinase MMP9, which can degrade MMRN2 under certain conditions and prevent its biological function from playing^25^. We observed that this phenomenon occurred when sMFI2 stimulated HUVEC. We found that when HUVEC was treated with sMFI2, the expression of MMP9 was decreased while the expression of MMRN2 was increased (Fig. 5D). To confirm the relationship between MMRN2 and MMP9, we constructed a MMP9 overexpression plasmid that was transfected into HUVEC by liposome (Fig. 5H and I). After overexpression of MMP9, the upregulation of MMRN2 in HUVEC induced by sMFI2 stimulation was attenuated (Fig. 5J and K), and the accompanying angiogenic function was also strengthened (Fig. 5L and M). In addition, we also unexpectedly found that with the overexpression of MMP9, sMFI2-induced HUVEC cell migration ability was also strengthened (Fig. 5N and O). This explains why the migration ability of HUVECs after co-culture with sMFI2 was weakened, probably due to the decrease in MMP9, and the migration-promoting ability of MMP9 was consistent in the literature^26–28^. In order to confirm the inhibitory effect of sMFI2 on angiogenesis in vivo, we injected 1 and 0 μg/ml Matrigel, respectively, into the subcutaneous of C57BL/6J mice and observed the status of angiogenesis after 7–10 days. Compared with the control group, the amount of angiogenesis in mice injected with 1 μg/ml was significantly reduced (Fig. 5P and R).

According to reports in the literature, sMFI2 has a strong ability to cross the blood–brain barrier^19,29–32^, and may affect the integrity of the connection between cells^33^. We hypothesized that since sMFI2 inhibits endothelial cell migration and angiogenesis, it might also affect the connections between endothelial cells leading to changes in vascular permeability and further inhibit tumor metastasis. To this end, we examined changes in cell–cell junctions after endothelial cells were co-cultured with recombinant sMFI2. The expression of ZO-1, P120, and N-cadherin, which are common intracellular connections increased to some extent after co-culture, indicating that the intercellular connections were not destroyed (Fig. 5Q). Permeability assay in vitro confirmed that the permeability of FITC-dextran in HUVEC co-cultured or not co-cultured with sMFI2 did not change (Supplementary Figs. S3C), and the number of trans-endothelial metastasis of A549 did not differ significantly in either case (Supplementary Figs. S3A and B). The above test indicated that although sMFI2 could inhibit angiogenesis, it did not affect vascular permeability, and there was little transfer of tumor cells across blood vessels.

### mMFI2 and sMFI2 are independent of each other in source and function

**Fig. 6:**
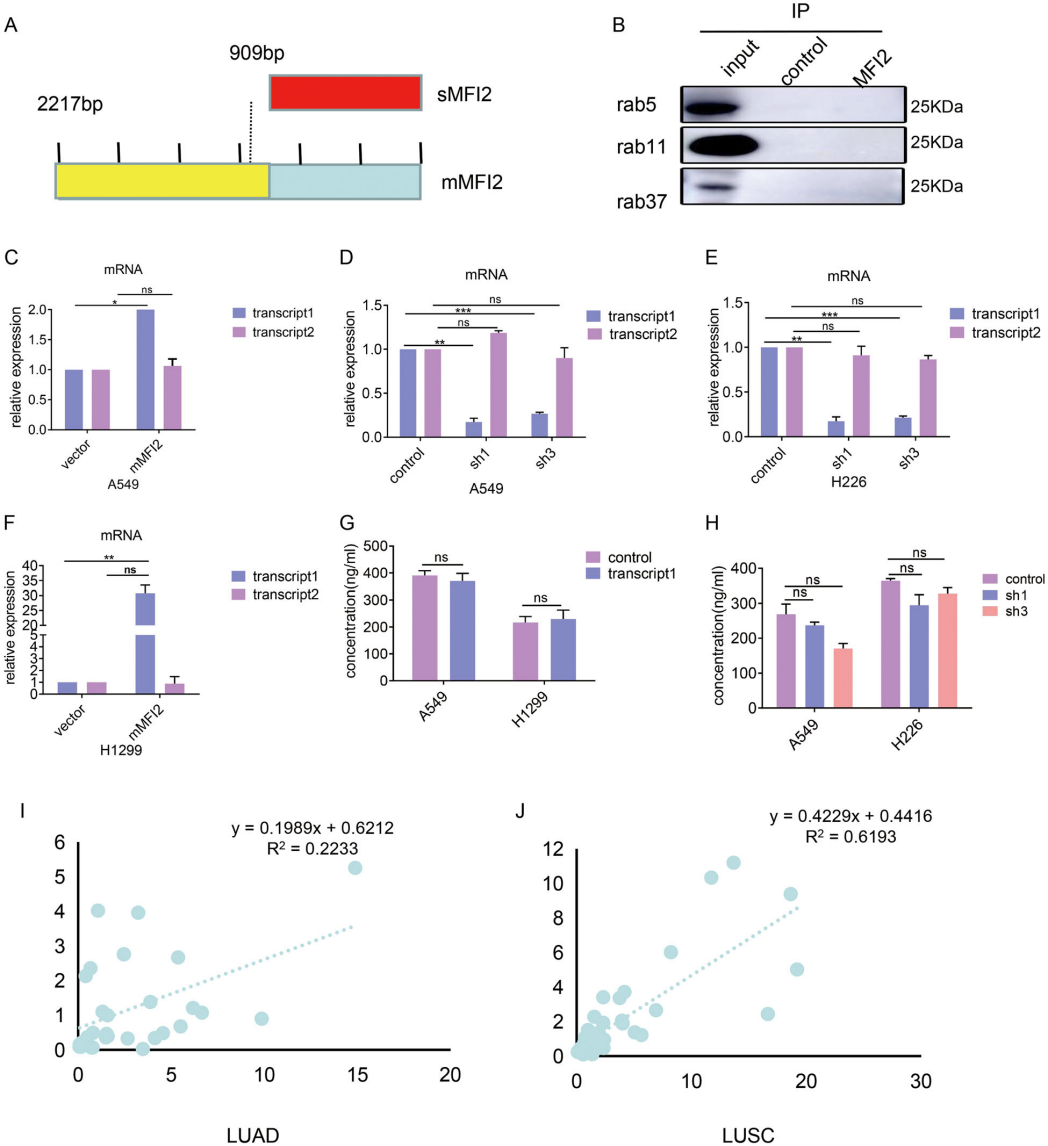
mMFI2 and sMFI2 are independent of each other in source and function. **A** Schematic diagram of mMFI2 and sMFI2 coding region (CDS). **B** Cellular lysates were extracted from A549 with overexpression of mMFI2 were subjected to immunoprecipitation with anti-mMFI2 antibody, followed by anti-rab5, anti-rab11, and anti-rab37 immunoblotting. **C–H** RT-PCR analysis of expression levels of mMFI2 (transcript 1) and sMFI2 (transcript 2) in cell clones A549, H1299, and H226 which had overexpression or knocking down of mMFI2. **I**, **J** Correlation analysis of expression levels of mMFI2 (transcript 1) and sMFI2 (transcript 2) expression in frozen tissues of lung adenocarcinoma and lung squamous cell carcinoma. Data are presented as the mean ± SD, *n* = 3. *p < 0.05, **p < 0.01, ***p < 0.001.

mMFI2 and sMFI2 are transcribed from the same gene MFI2, and there is a large overlap between these two. The CDS region of mMFI2 consists of 2217 bases encoding 738 amino acids, while the CDS region of sMFI2 consists of 909 bases encoding 302 amino acids (Fig. 6A). sMFI2 can be considered as a shortened form of mMFI2. We wanted to know if these two proteins interacted with each other in source and function. But we found there was no significant change in the mRNA of sMFI2 (transcript 2) after overexpression of mMFI2 (transcript 1) in cell lines A549 and H1299, as was the case after knockdown of mMFI2 (transcript 1) (Fig. 6C–F). Therefore, these two proteins are independent of each other at the transcriptional level.

According to the reports, mMFI2 has its own endocytosis function. mMFI2 and sMFI2 might only differ in structure by a GPI-anchored protein^21^. We assume that mMFI2 could be converted to sMFI2 by endocytic processing and re-secretion. Therefore, we used immunofluorescence to explore the distribution of mMFI2. We found that mMFI2 in the cytoplasm did express a significant aggregation after overexpression of mMFI2 (Supplementary Figs. S4A), indicating that mMFI2 did move intracellularly. To validate this phenomenon, we separately extracted the cytoplasmic components of mMFI2 overexpressing and non-overexpressing cell line A549, and then evaluated the cytosolic mMFI2 content by western blot. The result was consistent with the results of immunofluorescence. It indicated that in the case of ensuring consistent cell translation levels (consistent expression levels of GAPDH of the same number of cells) (Supplementary Figs. S4B), overexpression of mMFI2 could promote the accumulation of cytoplasmic mMFI2, and mMFI2 had a process from cell membrane to cytoplasm(Supplementary Figs. S4C). Therefore, we hypothesized that mMFI2 might be processed into a shortened form of sMFI2 after entering the cytoplasm, so that even if the two proteins are not related to each other at the mRNA level, they are linked at the protein level by post-processing modification. A protein whose full-length is to be shortened is generally subjected to preliminary processing by organelles such as endosomes and lysosomes. We guessed if there was a transformation from mMFI2 to sMFI2 and mMFI2 might partially present in the endosomes. Unfortunately, we confirmed by co-immunoprecipitation experiments that mMFI2 was not co-localized in the three common endosome markers rab5, rab11, and rab37 (Fig. 6B). In addition, we found that knockdown or overexpression of mMFI2 did not affect the secretion of sMFI2 (Supplementary Figs. S5A-5E). Thus, the conjecture that mMFI2 could form a shortened sMFI2 might be wrong. In order to clarify this problem, we re-extracted the cell culture supernatant of the mMFI2 overexpressing or knockdown cell clones and detected the secretion of sMFI2 by ELISA, there was no difference in the supernatant sMFI2 content (Fig. 6G and H). Therefore, no overexpression of flag was detected by cell supernatant after overexpression of mMFI2 labeled with flag (data was not shown). That is to say, the synthesis and secretion of sMFI2 were completely independent of mMFI2. Finally, we extracted frozen tissue samples from clinical patients and analyzed the expression levels of mMFI2 and sMFI2 transcripts by qPCR. Although the average transcripts of mMFI2 and sMFI2 in lung cancer patients were higher than those in adjacent tissues, these two transcripts were not highly correlated in terms of expression (Fig. 6I and J).

### mMFI2 and sMFI2 are associated with clinical outcomes

**Fig. 7:**
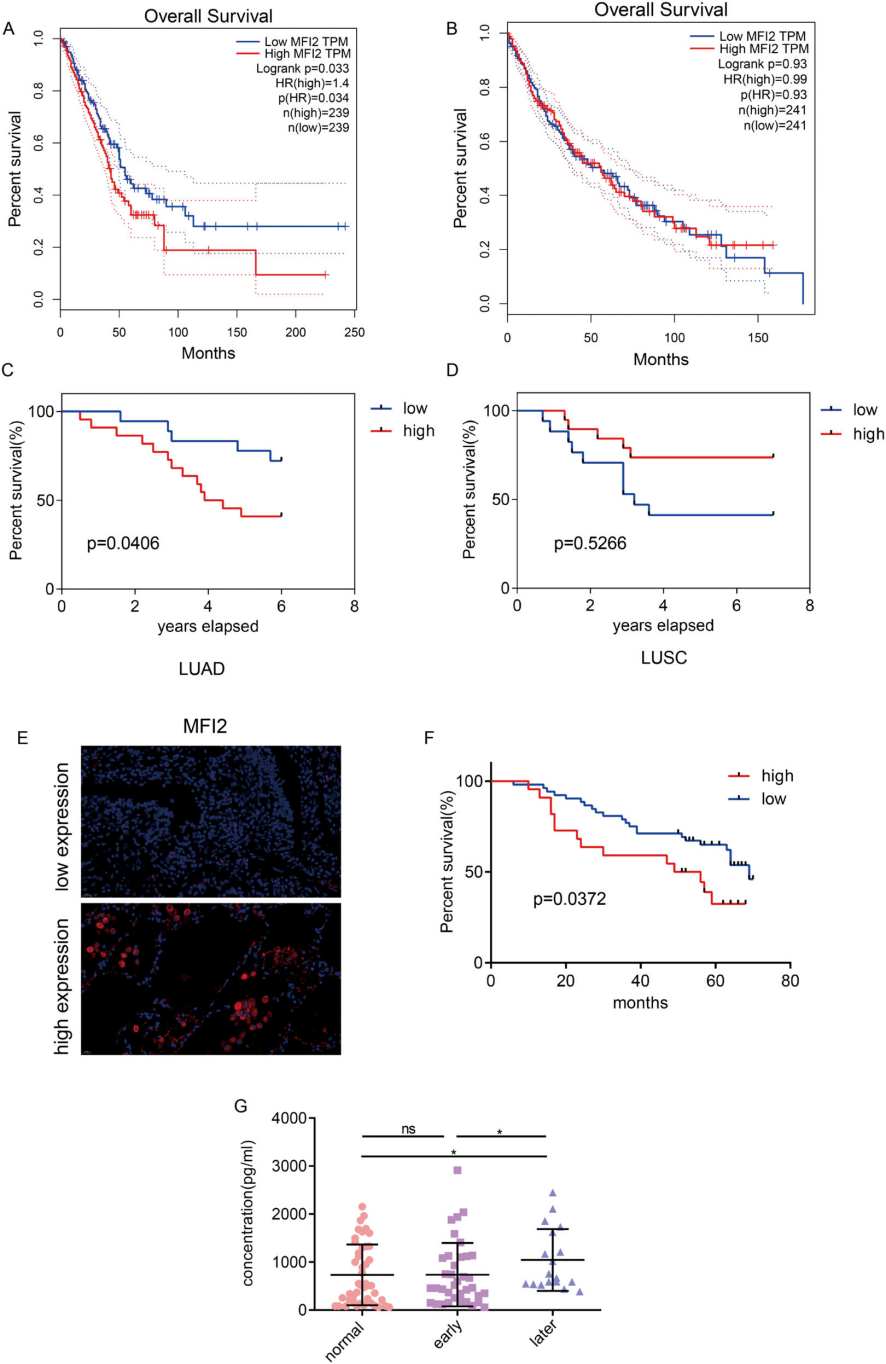
Both human mMFI2 and sMFI2 are associated with clinical outcomes. **A**, **B** Effect of mMFI2 on the prognosis of lung adenocarcinoma and lung squamous cell carcinoma in GEPIA database. **C**, **D** Relationship between expression of mMFI2 and survival rate in 34 cases of lung adenocarcinoma and 36 cases of lung squamous cell carcinoma. **E** Representative pictures of high and low expression of mMFI2 detected by immunofluorescence in 77 lung adenocarcinoma. Image taken under a ×400 microscope. **F** Relationship between expression of mMFI2 and survival time in 77 cases of lung adenocarcinoma. **G** Expression of sMFI2 in peripheral blood of 75 cases of lung adenocarcinoma and 55 normal cases detected by ELISA. Data are presented as the mean ± SD, *n* = 3. *p < 0.05, **p < 0.01, ***p < 0.001.

We first looked at the total MFI2 in the GEPIA database for lung cancer survival. Among 506 patients with lung adenocarcinoma, high expression of MFI2 resulted in lower overall survival (*p* value = 0.034) (Fig. 7A). However, among 495 patients with squamous cell carcinoma, there was no correlation between MFI2 and overall survival (*p* value = 0.93) (Fig. 7B). In addition, we collected 40 pairs of frozen tissues of lung adenocarcinoma and lung squamous cell carcinoma separately and analyzed the relationship between mMFI2 expression and prognosis of lung cancer. It was found that mMFI2 was highly expressed in lung adenocarcinoma and lung squamous cell carcinoma (Fig. 1F and I). There were 34 cases of lung adenocarcinoma and 36 cases of lung squamous cells were followed up in the later stage. The results of survival analysis were similar to those in GEPIA. That was, mMFI2 was associated with the survival rate of lung adenocarcinoma but not with lung squamous cell carcinoma (Fig. 7C and D).

Subsequently, we deparaffinized 77 paraffin-embedded lung adenocarcinoma blocks in the laboratory, created sections and performed immunofluorescence staining of MFI2. Among the 77 specimens, 22 had high expression of MFI2 and 52 had low expression. Figure 7E showed representative fluorescent images. Survival analysis of 77 samples also showed that MFI2 was a poor prognostic factor for lung adenocarcinoma (Fig. 7F). For the secreted protein sMFI2, we detected the content of sMFI2 in peripheral blood of 75 cases of lung adenocarcinoma and 55 normal subjects by ELISA. We found that the level of sMFI2 was relatively high in the late stage of lung adenocarcinoma, but since the content of sMFI2 was not significantly different between early patients and normal volunteers, it could not a good early diagnostic marker (Fig. 7G).

## Discussion and conclusion

Taken together, our study simultaneously analyzed the function and tumorigenic mechanisms of mMFI2 and sMFI2 encoded by the two transcripts of the gene MFI2 and interpret the relationship between mMFI2 and sMFI2. For MFI2 in lung cancer, the role it played by the diagnosis and treatment process has been explored at a deeper level.

MFI2 was discovered early in melanoma. Because of its homology to the sequence of transferrin^34^, it was natural to assume that this protein might involve in the biological behavior of the tumor by regulating iron metabolism. However, researches consistently confirmed that although MFI2 have a sequence of iron-binding like traditional transferrin, it does not transport iron^7,35–38^. Later research began to focus on its relationship with the biological behavior of tumors. However, the tumorigenic or antitumor function of MFI2, especially sMFI2, is controversial.

mMFI2 and sMFI2 are thought to be encoded by two different transcripts of MFI2^39^. Our results showed that mMFI2 could significantly promote the migration and invasion of lung cancer cells, consistent with features in melanoma^40,41^. Moreover, we found that the migration-promoting ability of mMFI2 is mainly achieved by downregulating KLF4, which is a well-known gene related to the stemness of tumor cells. Many tumor cells will develop EMT transformation during the process of transforming tumor cells into stemness, which also explains why mMFI2 can promote lung cancer metastasis.

We suspected that sMFI2 also had some functions similar to mMFI2, which promoted the metastasis of tumor cells. After treatment of A549 with sMFI2, the migration and invasion ability of A549 was indeed significantly enhanced. But in addition to the upregulation of N-cadherin, other markers of EMT such as snail, twist, and vimentin were also significantly upregulated, which indicated that TGF-β signaling pathway, Wnt signaling pathway, or Notch signaling pathway might be affected. sMFI2 can be secreted by tumor cells, which in turn promote the metastasis of tumor cells. This phenomenon can be regarded as an autocrine pathway. Since sMFI2 exerted its function as a secreted protein, we hypothesized that there might be a paracrine pathway. Therefore, we investigated whether sMFI2 affected endothelial cell function in lung cancer. We found that sMFI2 inhibited the migration of endothelial cells and subsequent angiogenesis. That is, sMFI2 has both cancer-promoting and antitumor effects. In lung cancer tissues, the content of mMFI2 is higher than that of sMFI2. Most of the sMFI2 we detected is still in the blood, and it has an inhibitory effect on blood vessels. Therefore, we believe that the overall effect of MFI2 is still pro-cancer. Inhibitor targeting mMFI2 should be able to play a better effect. In addition, sMFI2 lacks a membrane-bound motif relative to mMFI2, and we suspect that mMFI2 can be secreted after intracellular modification by auto-endocytosis. In other words, whether the content of extracellular secretory MFI2 protein could be affected by both sMFI2 and mMFI2? Although we could confirm that mMFI2 did translocate to the cytoplasm after overexpression, we did not find the presence of mMFI2 in endosomes or lysosomes, and the total amount of secreted protein did not change significantly after overexpression of mMFI2. In other words, mMFI2 and sMFI2 are encoded by different transcripts and then function through independent pathways.

To conclude, mMFI2 acts as a membrane-anchored protein and is a better therapeutic targeting molecule. Future research should be focused on the development of small molecule inhibitors of mMFI2 or specific neutralizing antibodies to evaluate its clinical significance in inhibiting tumor metastasis. sMFI2 has a high peripheral blood content in advanced lung cancer, which can be a good index for tumor staging, and needs to be verified by large samples.

## Materials and methods

### Clinical samples

Frozen tissues for RNA extraction were from the Cancer Hospital of Chinese Academy of Medical Sciences from October 2011 to November 2012. Paraffin specimen for immunofluorescence were from surgical patient in thoracic surgery of Cancer Hospital of Chinese Academy of Medical Sciences from January 2013 to August 2014. The blood sample used in the ELISA test was obtained from the Department of Clinical Laboratory, Cancer Hospital, Chinese Academy of Medical Sciences. Ethics approval was granted by the Committee for the Ethics Review of Research Involving Human Subjects of the Cancer Hospital of the Chinese Academy of Medical Sciences.

### Animal use

The lung colonization assay was performed using NOD/SCID mice purchased from Huafukang Bioscience (Beijing, China). Totally, 60 female mice aged 4–5 weeks were used. In addition, 30 female C57BL/6J mice from Beijing Huakang Biotechnology Co., Ltd were used for in vivo angiogenesis. Animal tests were described in the Supplemental Experimental Procedures. The animal studies were approved by the Animal Care and Use Committee of the Cancer Hospital of the Chinese Academy of Medical Sciences.

### Statistical analysis

Statistical analysis was performed using GraphPad Prism 6.0 and spss2.0. All data were presented as the mean ± standard deviation. Student’s *t*-test (two-tailed), one-way ANOVA, and the non-parametric Mann–Whitney U test was used to analyze the difference between groups. Pearson correlation analysis was used to explore the correlation between samples. Kaplan–Meier analysis was used to plot survival curves. *P* < 0.05 was considered statistically significant. All experiments were repeated at least two times.

For more details regarding “Materials and methods,” please refer to the Supplementary Materials.

## Supplementary information

Supplementary Information

Figure S1

Figure S2

Figure S3

Figure S4

Figure S5

Supplementary Figure Legends
