# Supplementary material for: The membrane-bound and soluble form of melanotransferrin function independently in the diagnosis and targeted therapy of lung cancer
Source: Cell Death Dis. 2020 Oct 30;11(10):933. doi: 10.1038/s41419-020-03124-2 (PMC7599248; doi:10.1038/s41419-020-03124-2)
Supplement: Supplementary file 1 — Supplementary Information [file 41419_2020_3124_MOESM1_ESM.docx]

## Supplementary Information

**The membrane-bound and soluble form of melanotransferrin function independently in the diagnosis and targeted therapy of lung cancer**

Yuanyuan Lei^1^, Zhiliang Lu^1^, Jianbing Huang^1^, Ruochuan Zang^1^, Yun Che^1^, Shuangshuang Mao^1^, Lingling Fang^1^, Chengming Liu^1^, Xinfeng Wang^1^, Sufei Zheng^1^, Nan Sun^1^**^＊,^** Jie He^1*^

**Experimental Procedures**

**Cell culture**

The lung cancer cell lines used in the study were as follows: A549, H226, H1299 and H460. Each cell line was identified by STR before use. The culture conditions of the lung cancer cell lines were as followings: all was placed at 37 °C, the humidity was constant, and the concentration of CO_2_ was maintained at 5%. The medium was recommended to use RPMI1640 with 10% fetal bovine serum and double antibody. The growth state of the cells was observed under a microscope every day, and two new complete media and double antibodies were replaced. Passage when the confluence of cell growth reaches 80%. Human umbilical vein endothelial HUVEC was cultured in DMEM high glucose medium, and other conditions were similar to lung cancer cell lines.

**RNA extraction and quantitative real time PCR (qRT-PCR)**

In the whole process of RNA extraction, the tips, pipettes, EP tubes and solutions used should avoid RNase contamination. All operations should be performed in an RNase-free environment. Also pre-cool the centrifuge to 4 °C. Total RNA was isolated with the standard Trizol-based protocol (Invitrogen). For the extracted RNA, use Nanodrop instrument to detect concentration and purity, directly used for reverse transcription. Reverse transcription was performed by Revert-Aid First-Strand cDNA Synthesis kit (Thermo Scientific), the whole process should be carried out on ice to avoid RNase contamination. The reagents and consumables used in the experiment must be free of RNA contamination. RT-PCR was performed on an ABI 7900HT Real-Time PCR thermocycler (Life Technologies). After the PCR reaction was completed, the base and threshold of the amplification curve were manually set. Export the data results and calculate the average of each group of Ct. Data analysis using 2^−ΔΔC^ method.

| Table S1 Primers used for real-time PCR | | |
| --- | --- | --- |
| Gene Symbol | Direction | Primer sequences |
| mMFI2(transcript1) | Forward | CAGTGCGTGTCAGCCAAGTC |
|  | Reverse | TTCCCCGCCGTGTAAATGT |
| sMFI2(transcript2) | Forward | AGCCGCCGAAAAACAGGA |
|  | Reverse | ACCAAACACCCGCACCAGA |
| MMRN2 | Forward | AGG CTTCCAGTACTAGCCTCTCT |
|  | Reverse | GGTAGGGGC ACCAGTTACG |
| MMP9 | Forward | GCGGTCCTGGCAGAAATA |
|  | Reverse | GTGAAGGCGCAGATGGTG |
| KLF4 | Forward | ACCT ACACAAAGAGTTCCCATC |
|  | Reverse | TGTGTTTACGG TAGTGCCTG |
| GAPDH | Forward | TGGGTGTGAACCATGAGAAGT |
|  | Reverse | CAGTGCGTGTCAGCCAAGTC |

**Western blot**

Protein were extracted with 1×RIPA lysate plus 1% volume of protease phosphoric acid inhibitor (100 ×).The kit for measuring protein concentration was Pierce BCA protein assay kit (Thermo Fisher).After measuring the concentration, add 1/4 volume of 5× protein loading buffer according to the volume of the cell lysate, mix well after shaking, centrifuge in a metal bath, and boil at 99 °C for 20 minutes to fully denature the protein. Then, configure the appropriate concentration of PAGE gel, keep the same amount of protein in each lane. The separated protein was transferred to a PVDF membrane, blocked with 5% skim milk powder for 1 hour, and then incubated with primary antibody overnight. The primary antibody types used in the experiment were as follows: MFI2(R&D Systems MAB8175), N-cadherin ( Abcam ), KLF4(Abcam), GAPDH(CST), twist(CST), snail(CST), Vimentin(CST), MMRN2(Abcam), MMP9(CST), rab5(CST), rab11(CST), and rab37(Abclonal). The blots were incubated with HRP-conjugated second antibody for two hours ,then performed ECL chemiluminescence color development.

**Cell proliferation assay**

The cell proliferation assay kit used in this study was the cell counting kit 8 kit purchased from Dojindo, Inc., Tongren Institute of Chemistry. When the cell fusion degree reached 70%, the cells were treated with trypsin digestion and then configured into a single cell suspension using complete medium., the cells were counted, and the cells were diluted to a concentration of 1-3 million/ml, fully mixed and then aspirated. 100μl was added to a 96-well plate and incubated at 37 ° C. At the same time point after 24 hours, 48 hours, 72 hours, and 96 hours, 110ul of complete medium containing 10ul of CCK8 was added to each well of a 96-well plate. Place in a 37 ° C and 5% CO2 cell incubator for 1.5-3 hours, then check on the machine, set 6-8 duplicate holes each time.

**Migration and invasion assays**

To compare the migration and invasion ability of different cells, we used the Migration and invasion chamber(Corning, 8.0-μm pores,3422/354480),Briefly, cells (5 × 104 for migration and1 × 105 for invasion) were seeded into the upper chamber in 200ul of serum-free RPMI 1640 medium, and 600ul of RPMI medium containing 20% FBS was placed in the lower chamber, after culturing for 24h, Erase the upper chamber cells , fix and stain the lower chamber cells, and count the number of cells that migrate to the lower chamber under an inverted microscope.

**Apoptosis assay**

The Annexin V Apoptosis Detection Kit from BD PharmingenTM was used for the apoptosis detection reagent in this study. At the same time, the nucleic acid dye propidium iodide was used to distinguish the fragments formed by the late necrosis and necrosis. The stably transfected cell line was inoculated into a six-well plate and cultured in a cell culture incubator at 37℃and 5% CO2 for 24 hours to achieve a cell fusion degree of about 60-80%.Incubate on ice for 5 minutes in the dark. Add 200ul of pre-formulated 1*binding buffer and transfer all liquid to the flow-loading tube for immediate testing.

**Protein chip**

The apoptotic chip was derived from RnDsystem (Catalog Number ARY009), which contains 35 apoptosis-related proteins. Capture and control antibodies have been spotted in duplicate on nitrocellulose membranes. Cell lysates are diluted and incubated overnight with the Human Apoptosis Array. The array is washed to remove unbound proteins, followed by incubation with a cocktail of biotinylated detection antibodies. Streptavidin-HRP and chemiluminescent detection reagents are applied, and a signal is produced at each capture spot corresponding to the amount of protein bound. Specific operation were done according to the manufacturer’s instructions.

**Transcriptome sequencing**

The transcriptome sequencing was completed by Beijing Boao Jingdian Biotechnology Co., Ltd. The sequencing platform was Illumina HiSeq. RNA was extracted from A549 and H1299 cells treated with or without mMFI2 overexpression. RNA integrity was assessed using the RNA Nano 6000 Assay Kit of the Bioanalyzer 2100 system (Agilent Technologies, CA, USA). Gene expression levels were determined based on FPKM (fragments per kilobase per million reads) values. Selection of differential genes by screening standard fold change≥2; *P*<0.05. Differentially expressed genes were identified based on their expression levels in different samples, and functional annotation and enrichment analysis were performed.

**Lentivirus production and construction of stable cell lines with overexpression or downregulation of mMFI2**

The overexpression lentiviral vector used in this study was constructed by Shanghai Jierui company. The construction of the lentiviral plasmid of shRNA was completed by Heyuan Bio (Shanghai) Co., Ltd. The shRNA sequences used for screening are as follows

| Table S2 shRNA target sequences for mMFI2 | | |
| --- | --- | --- |
| Gene Symbol |  | shRNA TargetSeq |
| MFI2 | sh1 | GGGCGAAGTGTACGATCAA |
|  | sh2 | GCAACAAGTGTGTGGGCAA |
|  | sh3 | CCGTCTTTGACAACACAAA |

**Transiently transfected siRNA and mmp9 overexpression plasmid**

SiRNA Transfection Reagent uses life technologies TM Lipofectamine RNAiMAX Reagent. The siRNA sequences used for N-cadherin and MMRN2 are as follows. The cells to be transfected should be prepared in advance, and the cell concentration should be adjusted to 80% of the operation.Configure transfection reagent according to manufacturer's instructions. Add the prepared transfection system to the 6-well plate at 250 ul/well. Finally, supplement to 2 ml / well with complete medium (without antibiotics). Mix the medium and mix the cells thoroughly with the transfection system. Incubate at 37 °C.RNA can be extracted after 24 hours of culture, and protein can be extracted after 48 hours. Overexpression plasmid against MMP9 was from Shandong Weizhen Biological Company

| Table S3 shRNA target sequences for N-cadherin and MMRN2 | | |
| --- | --- | --- |
| Gene Symbol |  | siRNA TargetSeq |
| N-cadherin | Si1 | GGGAGGTGTTGACTTTCAAAG |
|  | si2 | GCCGAAATACCCTGGAAATAG |
|  | si3 | GGCAAGGTATGCAAAGCAACT |
| MMRN2 | si1 | GCAGAACACTCAAGAGATG |
|  | si2 | CAGAATCTCCTCTCACTTT |
|  | si3 | GAATGTCATCTACTCCCAT |

**Immunofluorescence**

The cells with good growth state and confluency of 80% were digested according to the conventional method using trypsin enzyme to prepare a uniform cell suspension and counted, and the cells were diluted to 50,000 cells/ml, and 100ul of cell suspension was taken. Add to 8-well chamber slides (Biolex) for 24 hours. Then, cells were fixed by 4% paraformaldehyde, ruptured by 0.3% Triton X-100 for 5 min, blocked by 5% BSA for half an hour, and then the corresponding primary antibody was added overnight. The primary antibody used in this study was MFI2 (Stanta), N-cadheirn (Abcam), P120 (Abcam), ZO-1 (CST).According to the different species of the primary antibody, prepare the secondary antibody with 5% BSA solution, add 100ul of the second anti-solution solution to each well, and store at room temperature for 1 hour in the dark. After DAPI (ROEY）staining, it was observed under an inverted fluorescence microscope in a dark room and photographed.

**ELISA**

The kit for detecting sMFI2 was obtained from cloud clone corp (SEB677Hu). For cell supernatant, centrifuge with 1000g for 20min, then concentrate into a final volume of 250ul per 10ml with an ultrafiltration centrifuge tube (Sartorius VS15T91). For serum, first centrifuge with 1000g for 20 minutes, then dilute 20 times with PBS buffer, ie 10ul sample + 190ul PBS. Set the standard sample hole and the sample hole to be tested and the blank control hole, and add 100ul each hole, and make two duplicate holes for each, incubate for 1 hour at 37℃ ,and add detection A, detection B, substance and stop solution according to the instructions given by the manufacturer. Finally, run the microplate reader and conduct measurement at 450nm immediately.

**In vitro tubule formation**

Spread the melted matrigel on a pre-cooled 96-well plate on ice, add 60ul to each well, and place in 37℃incubator 30min, After the matrigel is completely coagulated, add a cell suspension of DMEM complete medium containing no or 10 μg/mL recombinant sMFI2. The number of cells per well is about 30,000, after 4-6 hours of culture. Photomicrographs were taken to observe the formation of vascular structures.

**In vivo Matrigel plug assay**

Growth factor-reduced Matrigel (BD Biosciences) mixed with or without 1ug/ml sMFI2 were injected subcutaneously into 6-week-old C57/BL6 mice. On day 7-10, the mice were sacrificed. Cut the skin around the matrigel in a clean bench, carefully peel off the skin on the matrigel with tweezers and remove it. Then remove the residual tissue and blood on the matrigel, observe the angiogenesis and take a picture.The formation of neovessels was indirectly determined by measuring the hemoglobin content using the Drabkin’s reagent kit (Sigma, St. Louis, MO) according to the manufacturer’s protocol.

**Co-immunoprecipitation Assay**

Pierce Classic Magnetic Bead Immunoprecipitation Kit was sourced from Thermo. mMFI2 overexpression and control group cells were added to the ice bath pre-cooled immunoprecipitation lysis/rinsing solution. 5minutes later, the lysate was centrifuged at 13000 g for 10 minutes, extracted the cell supernatant and measured the concentration. 500-1000ug of Total protein from the lysate supernatant of each sample was mixed with 2-10ug mMFI2 antibody and Incubated overnight at 4℃. The immunoprecipitated complex formed above was incubated with the pierce magnetic beads for 1 hour at room temperature, and the unbound magnetic beads and samples were washed away. Later, the protein was eluted with a low pH for subsequent western blot analysis.

**Chromatin immunoprecipitation assay (ChIP)**

ChIP assay was performed using Simple ChIP Enzymatic Chromatin Immunoprecipitation kit (9003, CST) according to manufacturer’s instructions. Briefly, Collect appropriate amounts of mMFI2 overexpressing cell line A549 and control cell lines. Perform nuclear processing and chromatin cutting. A cross-linked chromatin fragment containing 5-10ug of DNA was incubated with KLF4 antibody for overnight at 4℃. Finally, the chromatin was eluted from the magnetic beads, and the DNA was extracted from the spinnator. The obtained DNA was used for subsequent PCR quantitative identification to calculate the enrichment efficiency. Specific primers specific to the N-cadherin promoter region were required. (Forward: AGG CTT GGC AGT TTC CTT ACC C; Reverse: CCT GTC AGA GCA CAA GGC AAG A).

| Table S4. JASPAR predicted binding sites | | | | |
| --- | --- | --- | --- | --- |
| Transcription Factor | Score | Relative score | Stand | Binding sites |
| KLF4 | 10.4986 | 0.891197978 | + | CCACATCCTCC |
| KLF4 | 7.48806 | 0.824051657 | - | CCACGCGCTGC |
| KLF4 | 7.21069 | 0.817865182 | - | CCTCGCCCTGC |
| KLF4 | 6.91403 | 0.811248493 | - | TCCCACCCTTG |
| KLF4 | 6.66546 | 0.805704451 | + | CCTCACCCCCT |

**Mouse tail vein injection lung colonization**

In this study, an experimental tumor metastasis animal model was used. NOD/SCID mice were purchased from Beijing Huakangkang Technology company .A total of 50 NOD/SCID mice, 4-5 weeks old, weighing between 17 g and 20 g, all female, were raised in the heart center of the Chinese Academy of Medical Sciences. mice were placed at a constant temperature between 25°C; and 27°C; a constant humidity of 45% to 50%, freshly sterilized filtered air, dust free bacteria without a special pathogen breeding room. Animals were placed in a plexiglass box, each 5 mice. Then, the breeding box number was discharged on the ultra-clean biolayer flow rack, and the autoclaved water and material were freely ingested by the mice, and the autoclaved feed and pad are replaced every 3 days, the cage and the drinking bottle were kept to ensure strict adherence to aseptic procedures. While the NOD/SCID mice were subjected to an adaptation experiment environment, stable cell lines knocked down or overexpressed mMFI2 and respective control cell lines were expanded in vitro. When the degree of fusion of the cells in the culture dish reached 80 - 90% or more, the cells were digested with trypsin, a single cell suspension was prepared using the whole medium, and the cell concentration was adjusted to 10 ^7^ /ml using a cell counter. The cell suspension after completion of the counting was placed on ice and prepared to inject tumor cells into the tail vein of the mouse. Use a 1ml skin test needle for injection, mix well before aspirating the cell suspension, then pipette 0.5ml into the syringe tube, remove the air bubbles from the needle, and fix the mouse to a special device to make the tail exposed as much as possible. The tail vein of the mouse was wiped with a 75% alcohol cotton ball to allow the blood vessels to expand sufficiently, starting from the end of the tail vein. For the soft tissue surrounding, the injection should be stopped and the needle should be re-injected. During the injection process, the cells should be injected slowly and uniformly, not too fast, otherwise the mice were prone to heart failure. Within 48 hours after the tail vein injection, closely observe the state of the mouse within the hour, because this stage was an acute reaction period, there may be mice that died suddenly due to acute injury, and then observe the living state of the mice every 5 days. After 8 weeks, the mice were sacrificed by CO_2_ anesthesia, the lung tissue was completely dissected, and the blood on the lung tissue was washed with PBS buffer. The picric acid was fixed, photographed and embedded in paraffin and sectioned.
