# Supplementary figures and images for: The membrane-bound and soluble form of melanotransferrin function independently in the diagnosis and targeted therapy of lung cancer
Source: Cell Death Dis. 2020 Oct 30;11(10):933. doi: 10.1038/s41419-020-03124-2 (PMC7599248; doi:10.1038/s41419-020-03124-2)

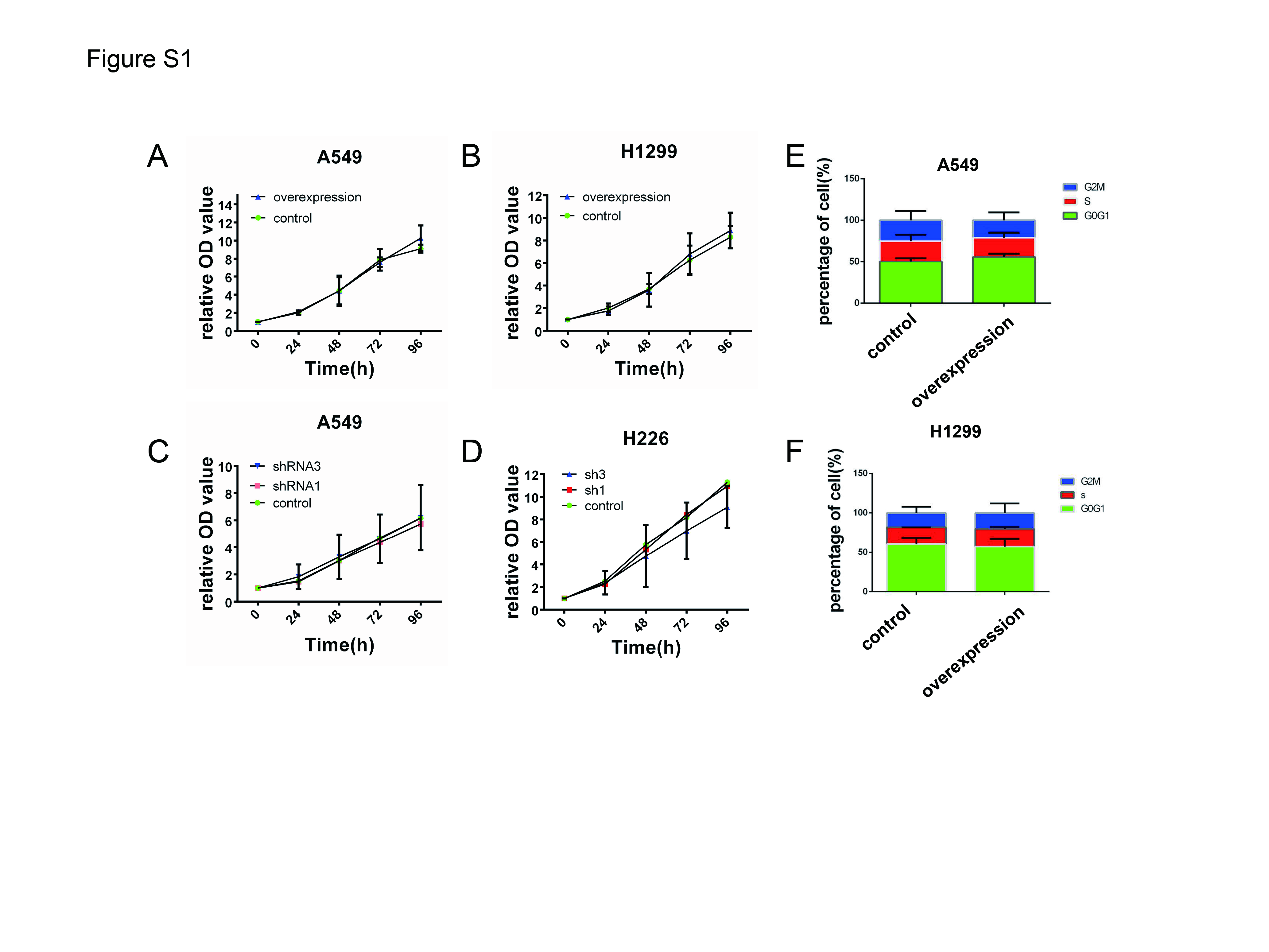

Supplement: Supplementary file 2 — Figure S1 [file 41419_2020_3124_MOESM2_ESM.tif]

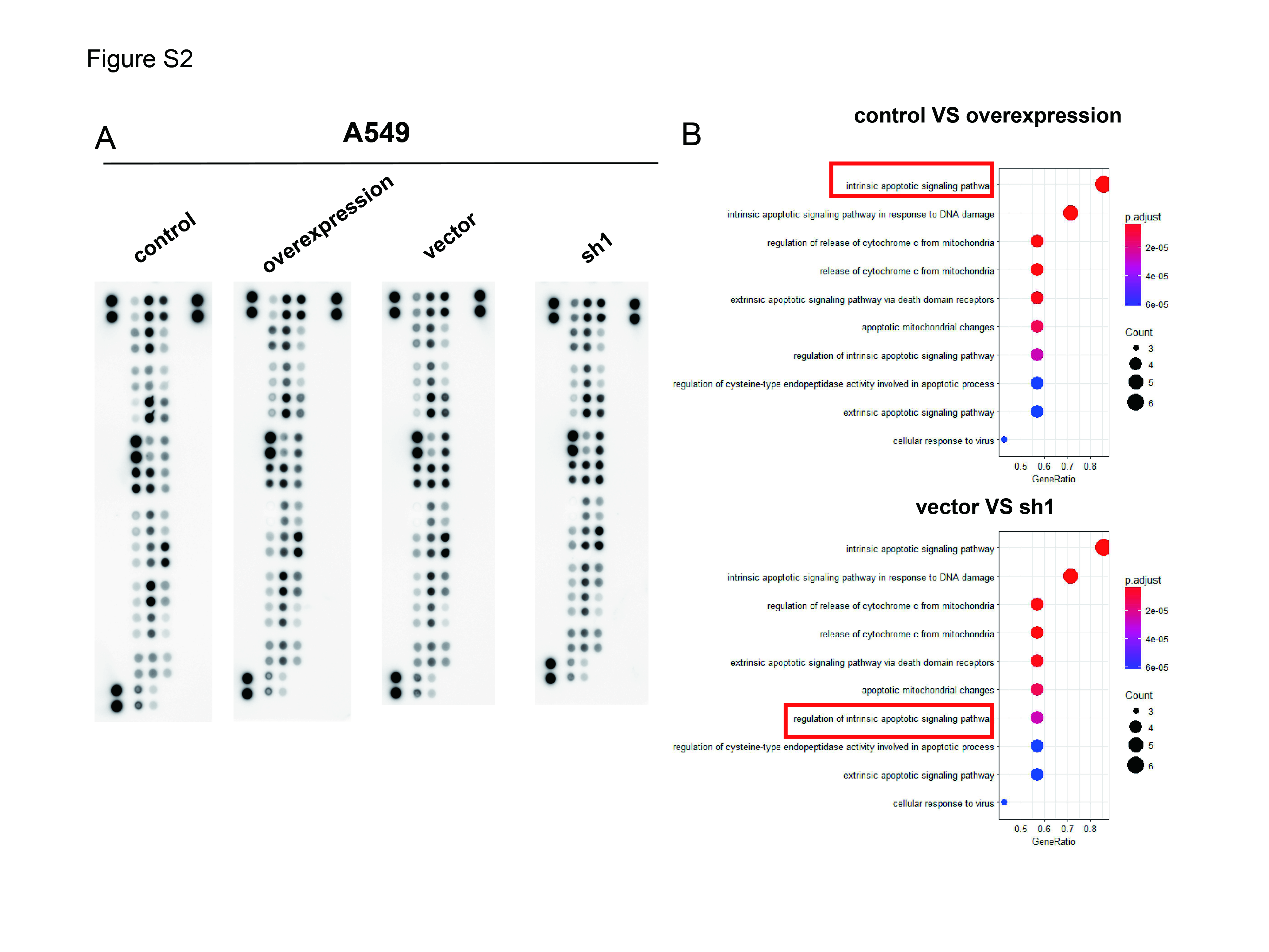

Supplement: Supplementary file 3 — Figure S2 [file 41419_2020_3124_MOESM3_ESM.tif]

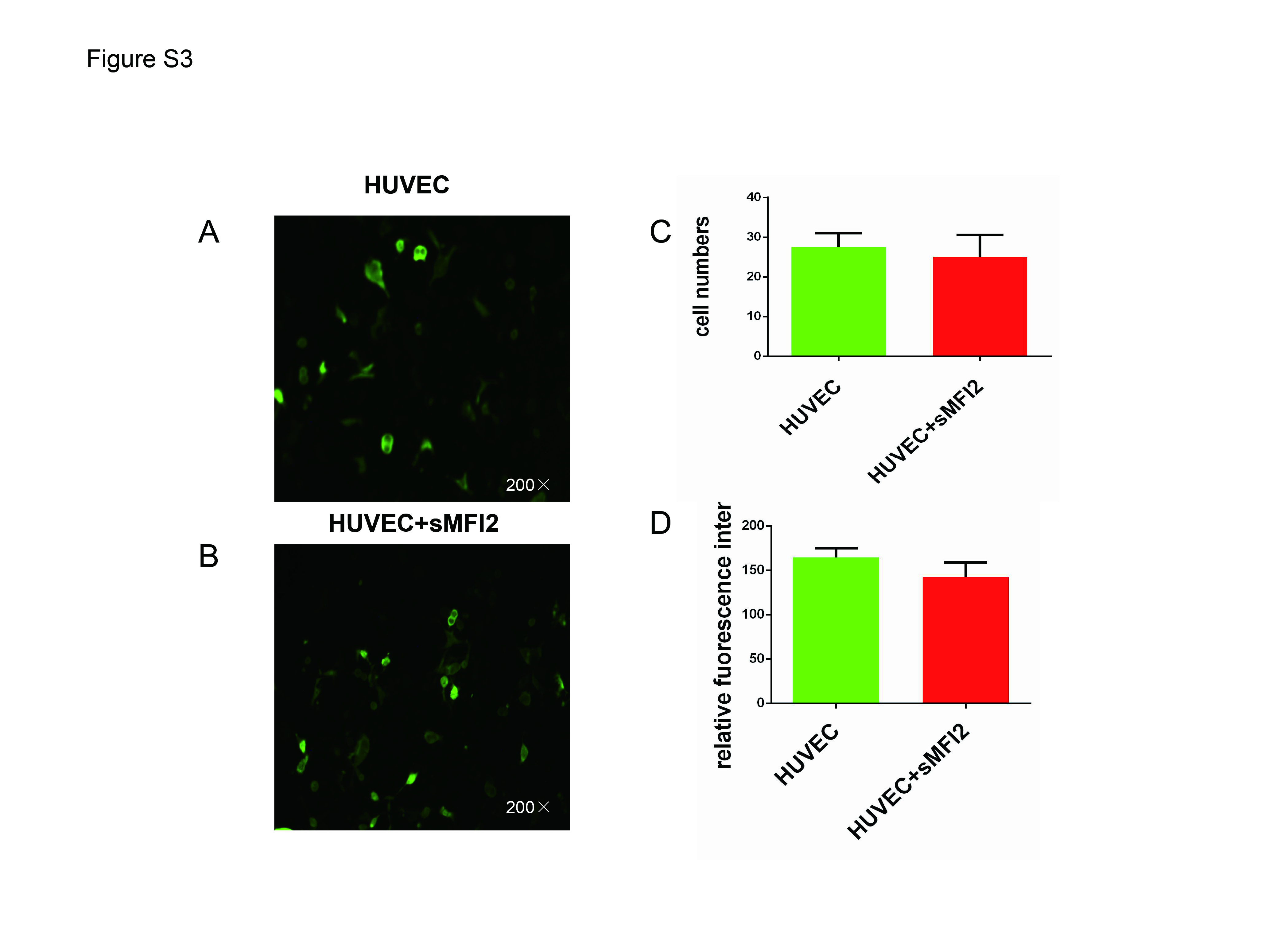

Supplement: Supplementary file 4 — Figure S3 [file 41419_2020_3124_MOESM4_ESM.tif]

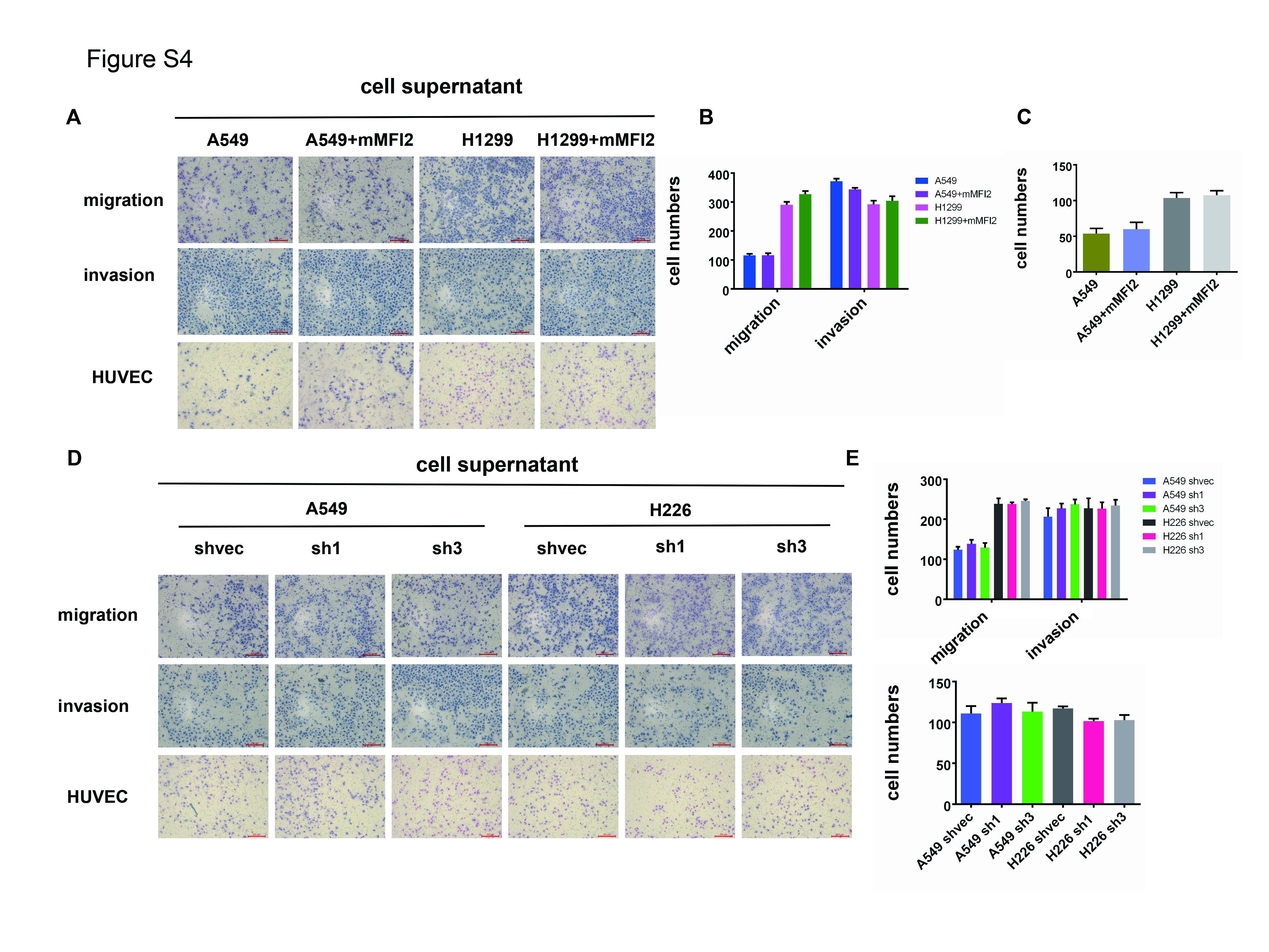

Supplement: Supplementary file 5 — Figure S4 [file 41419_2020_3124_MOESM5_ESM.tif]

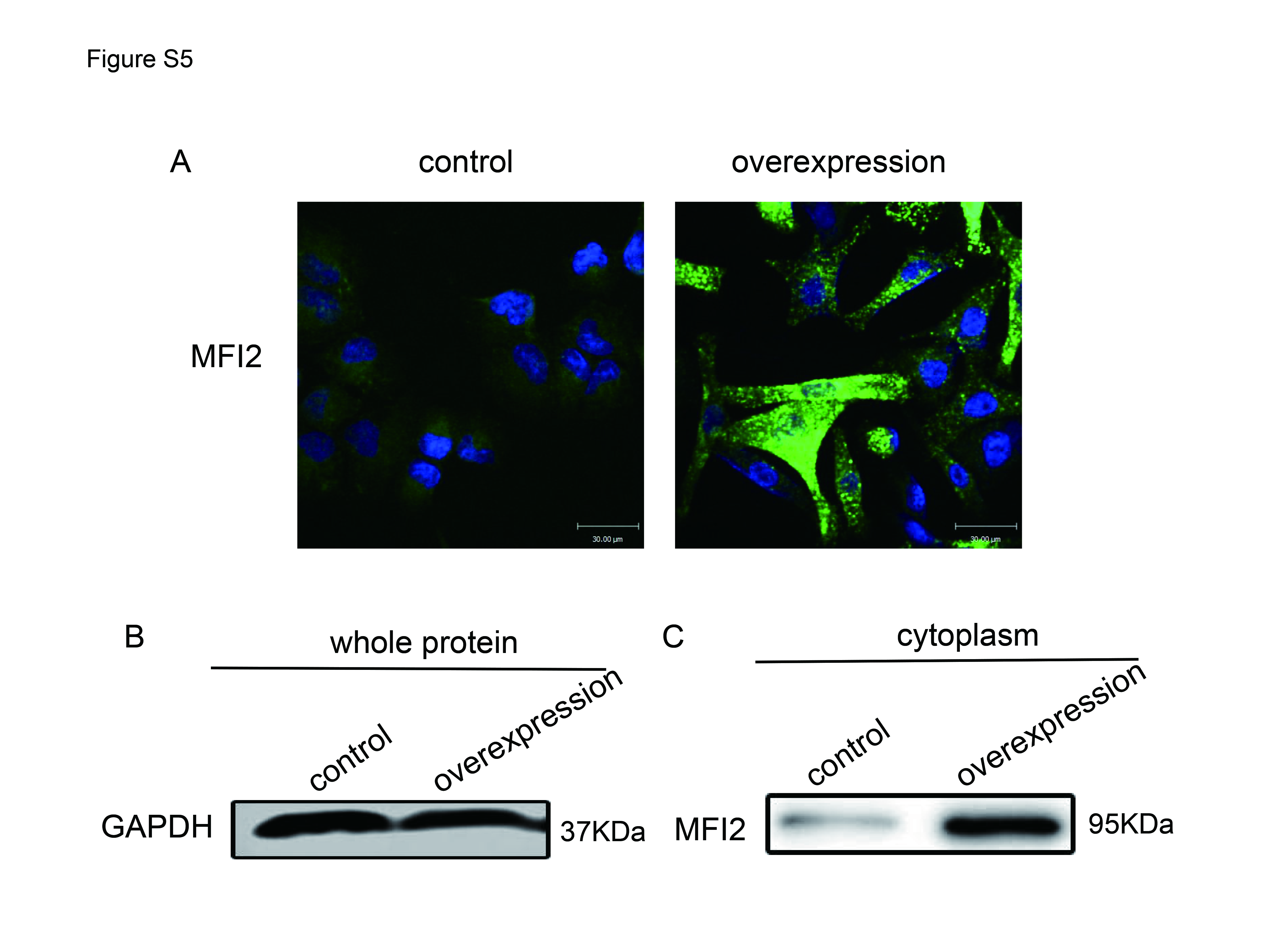

Supplement: Supplementary file 6 — Figure S5 [file 41419_2020_3124_MOESM6_ESM.tif]
