# Supplementary material for: The membrane-bound and soluble form of melanotransferrin function independently in the diagnosis and targeted therapy of lung cancer
Source: Cell Death Dis. 2020 Oct 30;11(10):933. doi: 10.1038/s41419-020-03124-2 (PMC7599248; doi:10.1038/s41419-020-03124-2)
Supplement: Supplementary file 7 — Supplementary Figure Legends [file 41419_2020_3124_MOESM7_ESM.docx]

**Figure S1. mMFI2 has no effect on tumor cell growth and cycle.** (**A-B**) The cell proliferation ability was compared between the indicated overexpression cell lines and negative control in A549 and H1299 cells. (**C-D**) The cell proliferation ability was compared between the indicated knock down cell lines and negative control in A549 and H226 cells. (**E**-**F**) Analysis of the periodic distribution of stably transfected overexpressing cell lines A549 and H1299 by flow cytometers. Data are presented as the mean ± SD, n = 3.

**Figure S2. Overexpression or knockdown of mMFI2 affects apoptosis pathway.** (**A**) Protein chip detection of 36 apoptosis-related proteins in stable overexpression、knockdown and their corresponding control cell line A549. Each dot represents a protein, and the color depth represents the level of expression. (B) Enrichment of apoptosis-related pathways. The red box indicates the signaling pathway that is a common change in overexpression and knockdown cell lines, namely the endogenous apoptotic pathway.

**Figure S3. sMFI2 has no effect on transendothelial transport of tumor cells and connectivity between endothelial cells.** (**A**-**B)** Capability analysis of lung cancer cell line A549 transfected with fluorescent plasmid GFP across endothelial cells in the presence or absence of sMFI2 (1μg mL−1). A: Representative images of control group. **B:** Representative images of co-culture group (**C**) Bar graph showing the numbers of A549 transcending endothelial cells in the control group and sMFI2 co-culture group. (**D**) Permeability analysis of HUVEC measured by relative fluorescence intensity of FITC–dextran flux (excitation 485 nm, emission 535 nm) treated with or without sMFI2s (1μg mL−1). Bar graph showing Fluorescence intensity changes between different groups. Data are presented as the mean ± SD, n = 3. *p< 0.05, **p <0.01, ***p < 0.001. blank: means no significance.

**Figure S4.** **Significant mMFI2 accumulation in cytoplasm after overexpression of mMFI2.** (**A**) Immunofluorescence showed mMFI2 was significant enriched in the cytoplasm after overexpression of mMFI2. (**B**)Western blot analysis of cytoplasmic proteins showed that cells overexpressing mMFI2 had significant mMFI2 enrichment in the cytosol. (**C**)The expression levels of GAPDH in the overexpression group and the control group were consistent, indicating that the translation levels of the two groups of cells were consistent.

**Figure S5 Knockdown or overexpression of mMFI2 does not affect the secretion of sMFI2.** (**A**) Changes in migration and invasion ability of wild-type cell A549 and migration ability of HUVEC in cell culture supernatants from stable cell lines A549 and H1299 overexpressing mMFI2. The above two rows are representative pictures of the migration and invasion abilities of wild-type A549, and the bottom row is a representative picture of the migration ability of HUVEC. (**B-C**) Bar graph shows quantitative indicators of migration and invasion of wild-type cell A549 and HUVEC. (**D**) Changes in migration and invasion ability of wild-type cell A549 and migration ability of HUVEC in cell culture supernatants from stable cell lines A549 and H226 with mMFI2 knocking down. The above two rows are representative pictures of the migration and invasion abilities of wild-type A549, and the bottom row is a representative picture of the migration ability of HUVEC .(**E**) Bar graph shows quantitative indicators of migration and invasion of wild-type cell A549 and HUVEC Data are shown as the mean ± SD n = 3. *p < 0.05, **p < 0.01, ***p <0.001. Blank means no meaning.

**Table S1** Primers used for real-time PCR

**Table S2** shRNA target sequences for mMFI2

**Table S3** shRNA target sequences for N-cadherin and MMRN2

**Table S4.** JASPAR predicted binding sites
